# Pen-type laser fluorescence device versus bitewing radiographs for caries detection on approximal surfaces

**DOI:** 10.1186/s13005-016-0126-9

**Published:** 2016-11-04

**Authors:** M. Bizhang, N. Wollenweber, P. Singh-Hüsgen, G. Danesh, S. Zimmer

**Affiliations:** 1 Department of Operative and Preventive Dentistry, University Witten/Herdecke, Alfred-Herrhausen-Str. 50, 58448 Witten, Germany; 2 Department of Operative and Preventive Dentistry and Periodontics, Heinrich-Hein University Duesseldorf, Moorenstr. 5, 40225 Duesseldorf, Germany; 3 Department of Orthodontics, University Witten/Herdecke, Alfred-Herrhausen-Str. 50, 58448 Witten, Germany

**Keywords:** Detection, Caries, Approximal, Pen-type laser fluorescence device, Sensitivity, Specificity, Radiography, Dentin caries

## Abstract

**Background:**

The accurate detection of approximal caries is generally difficult. The aim of this study was to assess the ability of the pen-type laser fluorescence device (LF pen) to detect approximal carious lesions in comparison to bitewing radiographs (BW).

**Methods:**

Three hundred forty-one tooth surfaces were diagnosed in 20 patients with an average age of 26.70 (±2.82) years. Each test tooth was sequentially assessed by a single calibrated examiner using visual inspection, BW, and the LF pen. Radiographs were used as the gold standard to calculate an appropriate cut-off.

**Results:**

Sensitivity, specificity and accuracy values for cut-off limits of 15, measured by the LF pen were compared using the chi^2^ test (McNemar test). For approximal caries at D3 level, the highest values of specificity and sensitivity were observed for the LF pen at a cut-off value of 15 (96.8 and 83.0 %) and for visual inspection (99.3 and 4.3 %).

**Conclusion:**

Within the limitations of this study, dentin caries on approximal surfaces could be detected equally well by the LF pen as by the bitewing radiographs. Therefore, the LF pen can be recommended as an alternative to radiographs for the detection of approximal caries in a regular dental practice setting.

**Trial registration:**

DRKS00004817 on DRKS on 12^th^ March 2013.

## Background

Caries detection on approximal surfaces is difficult. On one hand, visual examination for the detection of approximal caries lesions shows low sensitivity, and on the other, bitewing radiographs have been found to underestimate the lesion depth and are unable to reveal demineralisation in dentin [1]. Thus, in addition to the greatest drawback of exposure to radiation and the subsequent associated health hazards [2], radiographs do not show the correct size of the carious lesion [3]. Both the above-mentioned subjective diagnostic methods are of low sensitivity but high specificity for the detection of approximal caries [1]. As the determination of lesion depth possess a challenge in restorative treatment planning for most clinicians in daily clinical practice, radiography along with clinical findings is used as a routine diagnostic approach for approximal caries. Radiography of approximal caries increases the sensitivity of visual inspection and is, therefore, presently considered the gold standard for the detection of caries on approximal surfaces [4]. As mentioned above, the main disadvantage of BW is the exposition of the patients to ionizing radiation and the fact that it is technique sensitive [5]. Therefore, an alternative method to radiography, but with the same degree of diagnostic accuracy is desirable.

The pen-type laser fluorescence device (LF pen) (KaVo, Biberach/Riß Germany) has been developed to detect caries using the mechanism of fluorescence. The device produces a small laser with an excitation wavelength of 655 nm in the form of red light which measures the degree of bacterial activity. The reflection of light depends on the induction of fluorescence from bacterial porphyrins [6, 7]. A literature review carried out for the present study, revealed only two studies assessing the LF pen (one in vivo and one in vitro) for permanent teeth [4, 8]. The in vivo study found a fair positive correlation between laser fluorescence values and the radiographic scoring. Opened lesions analysed with their clinical lesion depths as gold standard, showed that there was a fair positive correlation to the laser fluorescence values and a moderately strong correlation to the radiographic scoring [4]. The in vitro study was able to demonstrate that the D3 threshold (dentin) ranged between 0.81 and 0.92 and that bitewing radiography showed an inferior performance compared to the LF pen [8]. Another study was able to establish the excellent intra/inter- examiner reproducibility for the LF pen on occlusal sites [9].

One study for detecting caries in primary teeth with the LF pen showed that simultaneously combined visual inspection with the LF pen and radiography increased sensitivity but decreased the specificity. The authors concluded that adjunct radiographic and LF pen methods offer no benefits for the detection of caries in primary teeth [10]. Another in vivo study examined the performance of the LF pen in comparison to conventional methods in detecting approximal caries lesion in primary teeth. This study found that the sensitivity for white spots was 0.20–0.21 for visual inspection, 0.16–0.23 for radiography and 0.16 for the LF pen and the specificity 0.95 for visual inspection, 0.99–1.00 for radiography and 0.94–0.96 for the LF pen [11]. The sensitivity for cavitation was 0.30 for visual inspection, 0.55–0.65 for the LF pen and 0.65–0.70 for radiography. The specificities for all methods were around 0.99 [11]. A further study showed that radiography and the LF pen achieved a similar performance in the detection of approximal caries lesion in primary teeth, however, the discomfort caused by visual inspection and the LF pen could influence the performance of these methods, since a higher number of false-positive or false-negative results occurred in children who reported discomfort [12]. On the other hand, an in vitro study found that a laser fluorescence device (LF), the LF pen and conventional methods perform similarly in detecting occlusal caries lesions in primary teeth. Thus, the study concluded that it is sufficient to employ visual inspection alone in clinical practice [13]. Visual inspection has been found to cause less discomfort than the other methods. Radiography and the LF pen presented similar levels of discomfort. Concurrently, older children reported high levels of discomfort with temporary separation, while younger children reported little discomfort with the LF pen [12].

The LF and LF pen have been demonstrated to achieve acceptable levels of performance in the detection of occlusal caries lesion in primary and permanent teeth [14–18]. Therefore, both can be considered to be suitable devices for occlusal caries detection [14, 19–22].

In vitro and in vivo studies observed no differences between the LF pen and BW performance in detecting approximal caries in primary teeth [11, 23, 24]. In permanent teeth, the LF pen showed better performance compared to radiography [8]. It necessary to undertake further in vivo investigations as results in the present literature show some disagreements.

## Methods

The aim of this study was to evaluate the LF pen for the detection of approximal caries lesion in permanent teeth in comparison to dental radiographs as the gold standard, for daily use in a regular dental practice setting.

Ethical approval for the study was obtained from the Ethical Board at the University of Duesseldorf (No. 3081), Duesseldorf, Germany. The first 20 patients who showed an interest, fulfilled the inclusion and exclusion criteria, and agreed to participate were asked to sign the consent form and were enrolled in the study. 8 males and 12 females participated in this study. In order to achieve an adequate power of 80 % and a defined significance level of 5 % (*p* < 0.05), the appropriate sample size was determined to be 20. Participants between 18 and 65 years of age, having at least 20 natural teeth with no current visual approximal caries lesion, periodontal disease, or other oral pathology were included in the study. Exclusion criteria were the presence of any systemic disease, pregnancy or breastfeeding, the use of fixed or removable orthodontic appliances, smoking and alcohol abuse.

Prior to commencement of the study both dentists carried out measurements on five subjects, not included in the study, to calculate the intra-examiner reproducibility. The intra-examiner and inter-examiner reproducibility levels ranged from 0.75 to 0.89 (good inter- and intra-examiner reproducibility levels).

After obtaining written consent from the subjects, the teeth were examined visually, BW and with the LF Pen. The mean age (standard deviation) was 26.7 (2.82) years. The mean DMFS (standard deviation) was 31.07 (15.46). The minimum and maximum of DMFS were 6 and 70. Thus, the subjects had moderate caries prevalence. A total of 561 posterior permanent approximal surfaces (both maxillary and mandibular) were examined in this study. 341 surfaces were included for the analysis of approximal surfaces. The study included 158 molar approximal surfaces, and 183 premolar approximal surfaces. 220 approximal surfaces were filled or without an approximal contact, and were thus excluded from the study. At the screening examination, radiographic evaluation of the carious lesions was carried out by taking BW.

One week after screening, the subjects received dental prophylaxis. The teeth were scaled using a Sonic Flex-Airscaler (KaVo Company, Biberach, Germany) and polished with a rotating soft latch-type Pro-Cup and Cleanic Prophy Paste (Kerr company, Washington D.C, USA). After polishing, the approximal surfaces were cleaned with dental floss (WaxedFloss, Johnson & Johnson Company New Brunswick, USA). Following this, the selected tooth was dried for at least 5 s with compressed air and examined under a standard operating light. Presence or absence of carious lesions was again recorded by visual examination using modified Ekstrand’s criteria and the LF Pen.

Modified Ekstrand’s criteria visual criteria [25]

0. No change in enamel translucency after air drying (> 5 s)
1. Opacity (white or brown) distinctly visible on the wet surface
2. Cavitation in dentine

The LF pen method was carried out using a probe tip for approximal surfaces (KaVo, Biberach, Germany). Prior to the examination, the LF pen was calibrated against a porcelain reference object and on the sound smooth surface of every tooth. After drying the tooth for 5 s with compressed air, the approximal area was measured by moving the tip under the tooth contact area, from the buccal to lingual/palatal side. The peak value was recorded [8]. Evaluation with the LF Pen was repeated three times. The maximum value for each measurement was registered and the mean value of the three readings was noted. LF pen values higher than 16 were used as a cut-off point to indicate the presence of a dentinal carious lesion [11].

Radiographs were used as the gold standard to calculate an appropriate cut-off. Bitewing projection geometry was employed to standardise the procedure for the digital images. Two BW were taken from each side for each subject. A Heliodent DS intra-oral x-ray unit with Sidexis intraoral sensors, aligned perpendicularly in a Rinn sensor holder (Sirona Company, Bensheim, Germany) at 60 kVp and 7 mA was used. The 5.6 × 36 mm XIOS sensor (APS-CMOS-Sensor, Sirona Company, Bensheim, Germany) was exposed for 0.12 s. The digital images were examined by the two dentists on a 18-in. CRT monitor (Multisync LDC 1990 SXI, NEC Corporation, Tokio, Japan) with the Sidexis software (version 1.61, Sirona Company, Bensheim, German) at X 2 magnification.

The following criteria were used for the radiographic examination [26]:

0. No radiolucency visible
1. Radiolucency visible in the outer half of the enamel
2. Radiolucency visible into the inner half of the enamel
3. Radiolucency visible in the outer half of the dentin
4. Radiolucency visible into the inner half of the dentin.

The investigator (NW) and another experienced dentist rated the BW under the same conditions. Presence or absence of dental caries on the BW was recorded. Decisions on the caries status and treatment of the cavity were made by both dentists together. In case of disagreement, the radiograph was discussed until a consensus was reached. After the final decision, the data of the subjects was either just recorded or recorded and subsequently the cavities treated. Radiographic codes of 3 and 4 were treated. Caries removal was carried out in the established cavities, thus enabling the clinical determination of caries level.

### Statistical analysis

All statistical analyses were performed with SPSS, version 19.0 for Windows (IBM/SPSS Inc. Chicago/IL, USA). Recordings of the visual examination and LF pen results were correlated with the gold standard BW to calculate the sensitivity, specificity and accuracy of the caries diagnostic techniques for approximal dentin caries. For the visual examination, the cut-off point was 1 for a sound surface and initial caries, and 2 for an established cavity. For the bitewing radiographs, scores 0 (healthy), 1 and 2 (enamel lesion) were registered as sound or initial carious lesions, and scores 3 and 4 (dentinal lesion) represented a cavity. ROC (receiver operating characteristic) analyses were also performed to determine a cut-off value. The Spearman rank correlation was determined so as to compare the caries level with radiology. Sensitivity, specificity and accuracy values for cut-off limits of 16 and 15, measured by the LF pen, were compared using the chi^2^ test (McNemar test). The tests were performed with α = 0.05 to analyse significant differences among the groups.

## Results

**Fig. 1:**
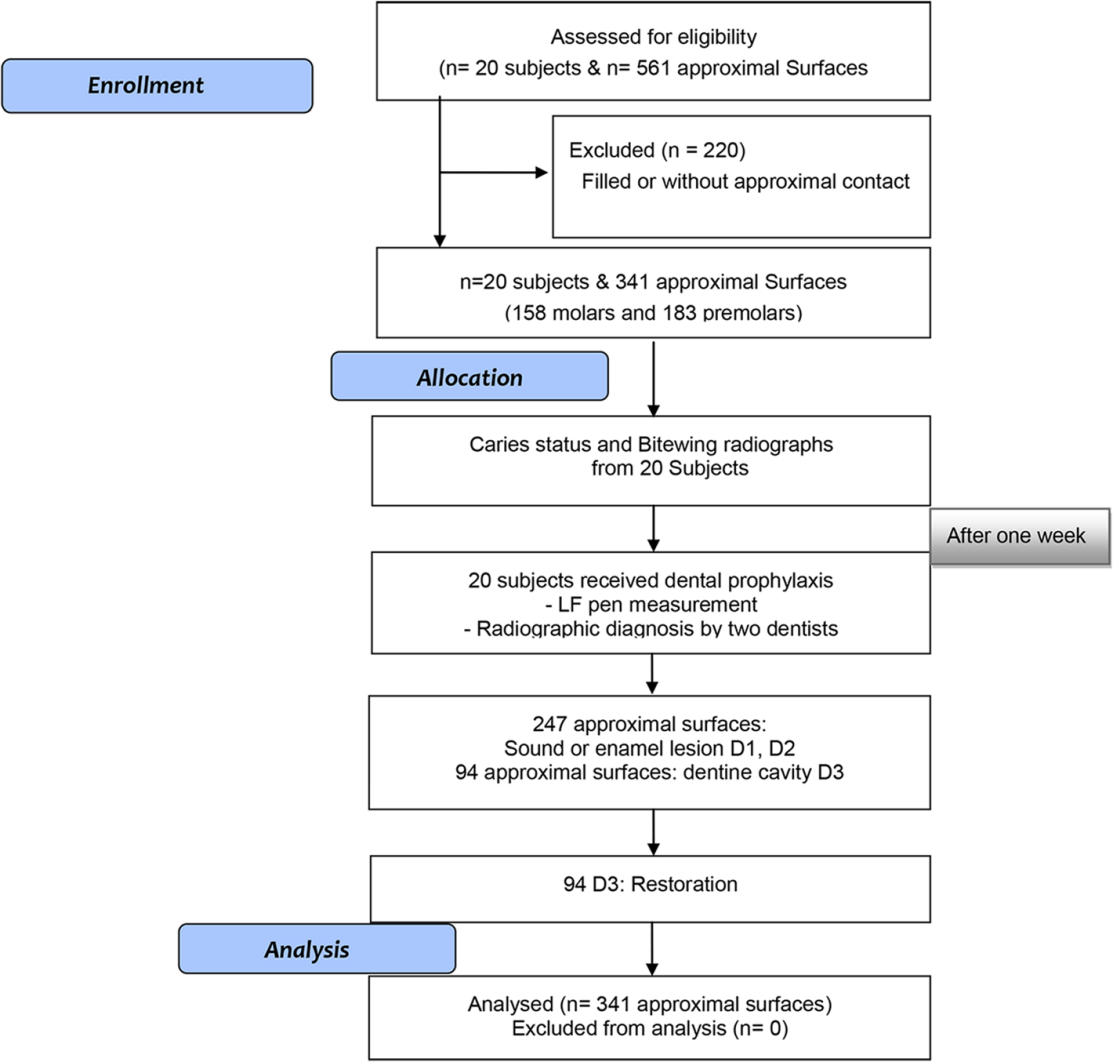
Flow diagram of the study

The patient drop-out-rate was 0 %. As the LF pen had failed to function in certain instances, 18 surfaces (5 %) were excluded from the analysis. The reason for this being, that on the examination day no functioning probe was available to replace the defective one. BW revealed enamel caries on 247 surfaces and 94 surfaces with caries extending into dentine (Fig. 1).

Cut-off points for the interpretation of LF readings for caries detection on approximal surfaces were determined after plotting values of sensitivities and specificities for the two different cut-off limits at dentine caries levels on a graph. The optimal cut-off limits for the LF pen was 15 for dentine caries in comparison to the manufacturer’s cut-off limits (16) (Table 1).

**Table 1:** Area under the ROC curves for LF pen for detection of approximal surfaces for cut-off limits 16 and 15

Method	Area	Standard error	*p*	Confidence interval (95 % CI)	
LF pen with cut off 16	0.883	0.026	0.002	0.833	0.933
LF pen with cut off 15	0.899	0.024	0.001	0.852	0.945

The values for molars and premolars have been presented separately in Table 2

**Fig. 2:**
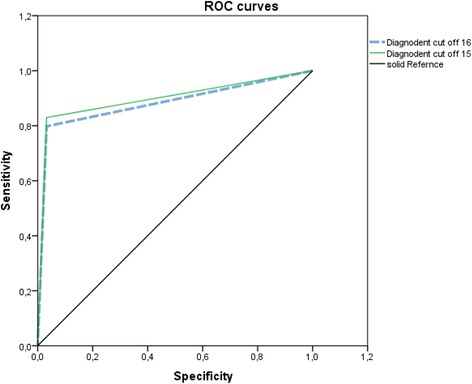
Receiver operating characteristics (ROC) for two different LF pen cut off limits for advanced dentine carious lesion on approximal surfaces

It was observed that the value of sensitivity was low for visual methods and high for the LF pen scores. Caries at the D3 level (dentinal lesion) showed values of specificity and sensitivity to be slightly higher for the LF pen at a cut-off value of 15 (Fig. 2). The Spearman rank correlation for approximal caries with radiography was 0.82 (LF pen 15), 0.79 (LF pen 16) and 0.12 (visual). The ROC (receiver operating characteristic) was significantly greater with LF Pen 15 and 16 compared to visual (Fig. 2).

## Discussion

All studies assessing caries radiographically show a marked increase in cavitation when the radiolucency reaches the outer half of dentine. In vitro studies have shown that the cavitation rate of inner enamel and outer dentine increases from 11 to 66 % and 65 to 100 % when the radiolucency has reached the outer half of dentine [27–29]. The use of BW as the gold standard in this study conformed with other studies having similar study designs [4, 8]. Furthermore, as several studies have shown LF to have a good reproducibility [8, 29, 30], it was not considered necessary to reconfirm the reproducibility of the LF pen. The examiner was found to have a good reproducibility. Visual inspection after tooth separation was not evaluated, due to non-acceptance on the subjects part, for reasons of discomfort [31] and time (two appointments). Moreover, in the daily dental practice setting, the decision to treat approximal caries is made without separation of the teeth. Thus, not separating the teeth prior to measurement with the LF pen also provided us with the knowledge of how functional the device is in a daily dental practice setting. Two authors have claimed that dental separation cannot be used as a validation method for permanent teeth because of low reliability [32, 33]. The study by Hintze et al. in 1998 showed, that two thirds of the surfaces under study were assessed as sound. After tooth separation, only a few (0.5–2.6 %) of these surfaces were found to be cavitated, thus showing a lack of reproducibility. The authors suggested that the tooth separation method cannot be used as a gold standard for validation of other diagnostic methods [33]. Ekstrand’s criteria for caries diagnosis is the most commonly used method in daily clinical practice, which most dentists agree upon to decide on the treatment need of a carious lesion [34]. In general, the studies that used a ranked diagnostic system such as that proposed by Ekstrand et al., which is also the basis of the ICDAS II criteria, had higher values for diagnostic parameters [35]. Similar to other study, we used the Ekstrand’s criteria for the present study [36].

A recent systematic review and meta-analysis with 75 studies, demonstrated that the fluorescence-based method for caries diagnosis tends to have similar accuracy for all types of teeth, dental surfaces or settings [37].

To evaluate the accuracy of a diagnostic method it is necessary to determine the validation method that expresses the true state of the disease. Sensitivity and specificity have been used in several in vitro and in vivo studies for the evaluation of the effectiveness of different caries diagnostic methods [19, 38–42]. Cut-off points for evaluation with the LF pen in primary teeth [11] and in permanent teeth [8] were obtained from an earlier study. Accordingly, readings higher than 5 were considered to be non-cavitated lesions, and measurements higher than 16, were considered to be cavitated. The results of this study revealed that the cut-off value of 15 had a slightly better validity than that of 16. In addition, the present study showed that the detection accuracy for approximal dentine caries was similar between the bitewing radiographs and clinical caries excavation of the same teeth (Table 2). This result was congruent with the result of other studies [4, 43].

**Table 2:** Sensitivity–specificity of visual and LF pen of approximal surfaces as compared with bitewing radiographs

	Sensitivity (95 % CI)	Specificity (95 % CI)	False positive	False negative	Accuracy (95 % CI)	Spearman (standard error)
Approximal surfaces (all)						
Visual	4.3 %^a,b^ (1.0–8.6)	99.3 %^a,b^ (98.3–100)	95.7 %	0.7 %	74.9 %^a,b^ (70.3–79.5)	0.15 (0.06)
LF pen (Cut-off 16)	79.8 %^a^ (71.4–88,2)	96.8 %^a^ (94.6–99,0)	20.2 %	3.2 %	92.1 %^a^ (89.2–95.0)	0.80 (0.04)
LF pen (Cut-off 15)	83 %^b^ (75.2–90.9)	96.8 %^b^ (94.6–99.0)	17 %	3.2 %	93.0 %^b^ (90.2–95.8)	0.82 (0.04)
Approximal surfaces (Molars)						
Visual	4.3 %^a,b^ (2.0–10.6)	98.4 %^a,b^ (96.1–100)	95.7 %	1.6 %	73.3 %^a,b^ (66.4–80.2)	0.13 (0.09)
LF pen (Cut-off 16)	73.9 %^a^ (60.3.87.5)	95.5 %^a^ (91.5–99.5)	26.1 %	4.5 %	89.2 %^a^ (84.1–94.3)	0.73 (0.06)
LF pen (Cut-off 15)	78.3 %^b^ (61.9–88.7)	95.5 %^b^ (91.5–99.5)	21.7 %	4.5 %	90.5 %^b^ (85.7–95.3)	0.75 (0.06)
Approximal surfaces (Premolars)						
Visual	4.3 %^a,b^ (1.56–10.16)	100 %^a,b^ (100–100)	95.7 %	0 %	76.5 %^a,b^ (70.4–82.6)	0.18 (0.06)
LF pen (Cut-off 16)	85.4 %^a^ (75.4–95.4)	97.8 %^a^ (95.3–100)	14.6 %	2.2 %	94.5 %^a^ (91.2–97.8)	0.86 (0.04)
LF pen (Cut-off 15)	87.5 %^b^ (78.1–96.9)	97.8 %^b^ (95.3–100)	12.5 %	2.2 %	95.1 %^b^ (92.0–98.2)	0.87 (0.04)

Different superscript letters express statistically significant differences within the same column for all approximal surfaces

Radiographic and LF pen methods increase the sensitivity of visual inspection in detecting approximal and occlusal carious lesions in primary and permanent teeth [1, 4, 15, 37]. The results of this study also showed that the LF pen increases the sensitivity of visual inspection in permanent teeth. These results were comparable to studies for primary teeth [11]. In contrast to these studies, an in vivo study demonstrated that adjunct radiographic and laser fluorescence methods offer no benefits in the detection of caries for approximal and occlusal caries in primary teeth in comparison to visual inspection alone [10]. However, this study had a low prevalence for approximal cavitated lesions with an intact marginal ridge (non-evident caries lesions) of 4.2 % for occlusal carious lesion and 5.25 % for primary teeth. The present study was for permanent teeth with a mean DMFS approximately 31 and a prevalence of approximal dentin caries of 27.6 % and of approximal sound/enamel lesions of 72.4 %. Mendes et al. speculated that the combined strategies could perform better in permanent teeth with a higher prevalence of non-evident caries lesions [10]. Nevertheless, most of the studies do not recommend the use of the LF or LF pen for permanent or primary teeth as the gold standard or sole diagnostic tool for occlusal caries detection [4, 39, 44, 45] or approximal caries detection [4] diagnostic tool to be used in combination with visual diagnostic techniques and/or radiography to assist the clinician when making operative decisions [46].

The first limitation of this study was that tooth separation was not carried out before visual examination in order to evaluate the approximal surface and thus to make a decision regarding preventive or operative treatment. The visual diagnosis was for the approximal area used in our study was not perfect, due to difficulty in viewing non-cavitated enamel lesions in this area [47], however it is functional method and provided the best alternative. In the present study, radiolucency in dentine was used as an indication for operative treatment of the lesion. This method is also the method of choice in daily clinical practice for detecting carious lesions and as an indication for treatment with a filling [48].

Use of only one examiner for the study, could be seen as another limiting factor of this study. This examiner was, however, calibrated with another experienced dentist prior to the commencement of the study on five subjects, who later did not participate in the study. The intra- and interexaminer reproducibility was found to be good.

## Conclusion

The results of this study show that the pen-type laser fluorescence device can be used for the detection of approximal carious lesions in permanent teeth similar to dental radiographs as the gold standard in a daily clinical practice setting.

Within the limitations of this study, the newly formulated cut-off limit of 15 instead of the cut-off limit of 16 for the pen-type laser fluorescence device provided a slightly higher validity, higher values of sensitivity and specificity, as compared to the gold standard BW. The high sensitivity and specificity of the LF pen make it suitable for the detection of approximal caries, in subjects with a similar caries experience, instead of radiographs for the clinician in a dental practice. However, further studies in larger sample size, with different caries experience, are needed to extend the standard usage of the LF pen for detection approximal caries.

## Abbreviations

BW: Bitewing radiographs
D3 level: Dentin lesion
DMFS: The decayed, missing, filled surfaces
LF pen: Pen-type laser fluorescence device
LF: Laser fluorescence device
